# Characterizing the tumor immune microenvironment of ependymomas using targeted gene expression profiles and RNA sequencing

**DOI:** 10.1007/s00262-023-03450-2

**Published:** 2023-04-19

**Authors:** W. de Koning, F. F. Feenstra, F. G. J. Calkoen, J. van der Lugt, L. A. Kester, D. A. M. Mustafa

**Affiliations:** 1 Tumor Immuno-Pathology Laboratory, Department of Pathology and Clinical Bioinformatics, Erasmus University Medical Centre, Rotterdam, The Netherlands; 2 Clinical Bioinformatics Unit, Department of Pathology and Clinical Bioinformatics, Erasmus University Medical Centre, Rotterdam, The Netherlands; 3 Princess Máxima Center for Pediatric Oncology, Utrecht, The Netherlands

**Keywords:** Tumor immune microenvironment, Targeted gene expression immune profiles, RNA sequencing, NanoString, Transcriptome analysis, Ependymoma

## Abstract

**Background:**

Defining the tumor immune microenvironment (TIME) of patients using transcriptome analysis is gaining more popularity. Here, we examined and discussed the pros and cons of using RNA sequencing for fresh frozen samples and targeted gene expression immune profiles (NanoString) for formalin-fixed, paraffin-embedded (FFPE) samples to characterize the TIME of ependymoma samples.

**Results:**

Our results showed a stable expression of the 40 housekeeping genes throughout all samples. The Pearson correlation of the endogenous genes was high. To define the TIME, we first checked the expression of the *PTPRC* gene, known as *CD45*, and found it was above the detection limit in all samples by both techniques. T cells were identified consistently using the two types of data. In addition, both techniques showed that the immune landscape was heterogeneous in the 6 ependymoma samples used for this study.

**Conclusions:**

The low-abundant genes were detected in higher quantities using the NanoString technique, even when FFPE samples were used. RNA sequencing is better suited for biomarker discovery, fusion gene detection, and getting a broader overview of the TIME. The technique that was used to measure the samples had a considerable effect on the type of immune cells that were identified. The limited number of tumor-infiltrating immune cells compared to the high density of tumor cells in ependymoma can limit the sensitivity of RNA expression techniques regarding the identification of the infiltrating immune cells.

**Supplementary Information:**

The online version contains supplementary material available at 10.1007/s00262-023-03450-2.

## Background

In the past years, the interest in molecular targeted therapy is rising. However, it has primarily focused on genomics. Transcriptome analysis is a high-accuracy strategy to define the tumor immune microenvironment (TIME) of patients based on RNA [1]. The TIME is known to be related to cancer progression and therapeutic outcomes [1]. Transcriptome analysis is a useful technique to study different cellular processes, such as immune responses and cell types that are present in the TIME [1].

Over the last decades, RNA sequencing is the most used transcriptomic analysis to understand genomic functions [2, 3]. Fresh frozen (FF) samples are used to sequence the whole transcriptome via amplification. RNA sequencing can be used to find biomarkers since it does not require specific probes [4]. Profiling tumors with RNA sequencing can provide insights regarding classification and progression. The four main steps are [1] mRNA transcript fragmentation, followed by random primer binding, [2] cDNA synthesis via reverse transcription of the mRNA, [3] tagging the ends with a phosphate group and poly(A) tail and [4] ligation of adapters which enables PCR amplification and sequencing. Although this method is the most used, it has several disadvantages. High-quality RNA is needed for the amplification step of RNA sequencing, and due to the low signal/noise ratio, some transcripts are difficult to detect [4]. Furthermore, analyzing these RNA sequence data could be expensive, and it needs technical and bioinformatical skills [5, 6].

In contrast to RNA sequencing, NanoString gene expression analysis is targeted and measures a selected set of genes. In addition, NanoString targeted gene expression analysis does not require amplification, which reduces workflow errors and increases reproducibility [7, 8]. Formalin-fixed paraffin-embedded (FFPE) samples are used to perform targeted gene expression analysis [7]. This method is used in diagnostics, and it can measure up to 800 genes and even low counts [6, 7]. NanoString’s nCounter is a hybridization-based method, instead of amplification-based RNA sequencing, to detect RNA transcripts [9]. Different gene panels can be ordered via NanoString, for instance, the PanCancer Immune Profiling Panel [10]. NanoString targeted gene expression analysis is known to be a robust method with minimal background. This method can identify genes regardless of low-quality RNA or less-than-ideal FFPE preparation before gene expression analysis [5, 6]. In addition, only a little amount of RNA (approximately 25 ng) is needed for this method [5]. The workflow is more user-friendly than the workflow of RNA sequencing since it does not include library preparation [4]. Furthermore, NanoString provides software to analyze your RNA data, which is called nSolver™. This nSolver™ software can identify cellular processes and cell types based on RNA expression levels [11].

Ependymomas account for 8–10% of pediatric brain tumors, and the standard therapy consists of surgery (as radical as possible) and radiation therapy [12–14]. However, this treatment remains unchanged for the past two decades [12–14]. Although 50–70% of the tumors are successfully treated with surgery and radiation therapy, no standard care therapy is available for recurrent or persistent ependymomas resulting in a dismal prognosis for these patients [15]. In addition, recurrence occurs in almost 50% of ependymoma patients [12]. Therefore, there is a need for alternative treatments for patients with ependymoma. Nine different subgroups of ependymomas can be defined based on the location of the tumor, genetics, and epigenetic DNA methylation [12, 16]. The focus of this study is on posterior fossa group A (PF-A) ependymomas, which occur in children (aged 0–18 years). PF-A ependymomas are located in the cerebellum and arise from regional radial glial-like cells [17]. PF-A ependymomas are known to be aggressive due to low mutation burden and high activation of several pathways, such as proliferation and angiogenesis, leading to a poor prognosis [15]. Another aggressive ependymoma subgroup is located in supertentorium (ST). In this study, both subgroups were included for analysis.

Knowledge of the immune system has great importance for treating cancer patients with immune therapies. In recent years, the Food and Drugs Administration (FDA) has approved immune checkpoint inhibitors for solid tumors in adults and chimeric antigen receptor (CAR) T-cell therapy for children with leukemia [13]. However, immune therapies are challenging in brain tumors since the brain is protected by the blood–brain barrier (BBB), which is known to limit the infiltration of therapies [18]. To date, little is known about the TIME of ependymomas [19, 20]. The TIME of pediatric CNS tumors tends to be immune suppressive [21, 22], and there are indications that the TIME of ependymomas might also be immune suppressive or ‘cold’ TIME, indicating a lack of tumor-infiltrating T-cells [23].

Previous studies have demonstrated the high correlation between RNA sequencing and the NanoString gene expression analysis [6, 24, 25]. However, none of these studies focus on specific cancer tissue, nor did the studies investigate the TIME. This study aims to highlight the (dis)concordance in the identification of the TIME in a cold tumor, like ependymomas, using gene expression data generated by measuring bulk RNA with the two most common techniques: targeted gene expression and RNA sequencing.

## Material and methods

### Sample collection and processing

Six formalin-fixed paraffin-embedded (FFPE) and fresh frozen (FF) ependymoma tumor samples (*n* = 2 ST, *n* = 4 PF-A ependymomas) from the same tumor were collected at the Princess Maxima Center (Utrecht, the Netherlands). Each sample is a primary tumor before either radiation therapy or chemotherapy and was collected between 2019 and 2020. The BioBank committee of the Princess Maxima Center approved the application. In addition, this study is in line with the declaration of Helsinki.

### *NanoString immuneprofiling*

Six FFPE ependymoma tumor samples were collected. Before RNA isolation the samples were sectioned with HM 340E Electronic Rotary Microtome. The whole sample was used to include the TIME. First, samples were put at − 15 °C. Sections of 10 µm were cut to finally have 100–200 µm of each sample. These sections were put in a 42 °C water batch and afterward added to a slide. The slides were dried overnight before deparaffinization was performed. The nCounter^®^ PanCancer Immune Profiling Panel was used for NanoString targeted gene expression analysis of six FFPE ependymoma tumor samples. This panel consists of 730 genes and 40 housekeeping genes targeting both innate and adaptive immune cells, and different pathways such as checkpoint signaling and antigen processing, which are considered an important part of the TIME (10). The total RNA was isolated from tumor tissue using the RNeasy^®^ FFPE isolation kit (Qiagen, Leiden, the Netherlands). The RNA quantity and quality were measured using the Agilent 2100 BioAnalyzer (Santa Clara, CA, USA). RNA concentration was corrected to include fragments ≥ 300 bp. For each sample, 300 ng of RNA was hybridized with the PanCancer Immune Profiling probes for 17 h at 67 °C, following the manufacturing procedure (NanoString Technologies Inc., Seattle, WA, USA). The nCounter^®^ FLEX platform was used to wash the extra probes, and genes were counted by scanning 490 fields of view (FOV).

### Data analysis of NanoString immune profiling

The raw data of gene counts were uploaded to the nSolver™ Data Analysis software (version 4.0, NanoString, Seattle, WA, USA). Genes that had an expression level below the average count of the negative controls plus two standard deviations are considered undetected. The gene counts were normalized using the most stable housekeeping genes using the Advanced Analysis module (version 2.0) of nSolver™.

### RNA sequencing

Six fresh frozen (FF) ependymoma tumor samples were collected. For all samples, 300 ng input material was collected and processed using RNA sequencing transcriptome analysis at the Princess Maxima Center. The RNA sequencing library was prepared with the Roche KAPA hyperprep kit, including the amplification step. The library was sequenced with Illumina Novaseq 6000 using 2 × 150 bp sequencing. The paired-end sequencing reads were aligned to the human reference genome (GRCh38.p12, hg38) using STAR [26] and annotated with transcript annotation (Gencode Release 31). Transcript quantification was performed using Subread featureCounts [27]. The counts were gene length trimmed mean of M-values (geTMM) normalized afterward [28].

### Housekeeping genes

The PanCancer Immune Profiling Panel includes 40 housekeeping genes. These housekeeping genes were tested in the RNA sequencing and NanoString results for their stability using the geNorm algorithm [29]. The algorithm identified a minimum number of genes required to calculate a normalization factor as a geometric mean, which was used in the NanoString normalization. The most stable housekeeping genes from both techniques were compared by looking at the variation in the gene expression between samples.

### Cell identification

To characterize the immune cell infiltration, gene markers were identified [30]. The gene markers were selected by calculating the pairwise similarity between all pairs of candidate marker genes that were above the detection limit in at least 50% of the samples. The gene pairs with a pairwise similarity above 0.6 were selected to describe the immune cells. Each immune cell type needed at least two unique genes. The abundance of the immune cell types is the average expression value of their corresponding marker genes.

The identification of tumor-infiltrating leukocytes (TILs) with the RNA sequencing data was performed using the CIBERSORT method [31] and the MCP-counter [32]. CIBERSORT combines support vector regression with prior knowledge from expression profiles from purified leukocyte subsets to estimate the immune composition. The validated leukocyte gene signature matrix (LM22) including 547 marker genes was used to quantify 22 human hematopoietic subsets. The absolute mode was run together with 1000 permutations without quantile normalization as recommended by the developer [31]. MCP-counter is a method that allows absolute abundance calculations of eight immune and two stromal cell populations with the use of transcriptomics markers [32].

Immunohistochemistry was performed at the UMC Utrecht Pathology department on 5-μm FFPE tumor tissue sections using a Ventana Immunostainer.

### Statistical analysis

The official gene symbols approved by the HGNC were used to identify matches between the NanoString and RNA sequence datasets. The Pearson correlations of the samples between the two platforms were computed with R software, version 4.1.1 [33]. To detect the genes that differ the most from the expected correlation, a linear model for each sample was created and the residuals were reported.

## Results

### Patients’ characteristics

A total of six patients were included that were diagnosed with ependymoma. The FFPE samples and fresh frozen samples of the 6 patients were collected, and RNA was successfully isolated. The patients were between 0 and 13 years old. (Mean age is 4 years.) All clinicopathological characteristics are summarized in Table 1.

**Table 1:** Clinicopathological characteristics at the time of diagnosis from the six patients included

Sample	Age	Sex	Diagnosis	Tumor location	Resection location	Molecular subgroup	Fusion status
EPN-01	13	F	WHO III	ST	Supratentorial right frontal		RELA fusion*
EPN-02	2	F	WHO III	ST	Supratentorial left frontal		RELA fusion
EPN-03	1	M	WHO III	PF	Fossa cranii posterior	Group 1	PF-A
EPN-04	2	M	WHO III	PF	Fossa cranii posterior	Group 2	PF-A
EPN-05	2	M	WHO III	PF	Fossa cranii posterior	Group 2	PF-A
EPN-06	3	M	WHO III	PF	4th ventricle	Group 2	PF-A

*Sample clusters as RELA but a ZFTA or RELA fusion was not detected

### Characterization of ependymoma using NanoString

Of the 770 genes included in the nCounter^®^ PanCancer Immune Profiling Panel of NanoString Technology, 752 genes were detected in at least one sample. The total number of genes detected per sample was comparable between the six samples. However, the number of the detected genes was lower in three samples, namely EPN-04, EPN-05, and EPN-06 (Supplementary Fig. 1A).

### Characterization of ependymoma using RNA sequencing

In total 49,136 unique RNA molecules were detected in at least one sample by RNA sequencing out of the 58,804 features described in the transcript annotation. This included 19,051 protein-coding sequences (Table 2). The protein-coding sequences are the only features that can be compared to the 770 genes included in the NanoString panel.

**Table 2:** Number of unique RNA molecules detected in at least one sample per RNA molecule category by RNA sequencing

Category	Frequency
Protein coding	19,051
Long noncoding RNA	14,696
Processed pseudogenes	10,219
Others	2860
Small nuclear RNA	1238
Micro RNA	1072

The total number of features detected per sample was comparable between the six samples. However, the number of detected features was the least in sample EPN-05 (Supplementary Fig. 1B).

### The expression stability of the housekeeping genes

**Fig. 1:**
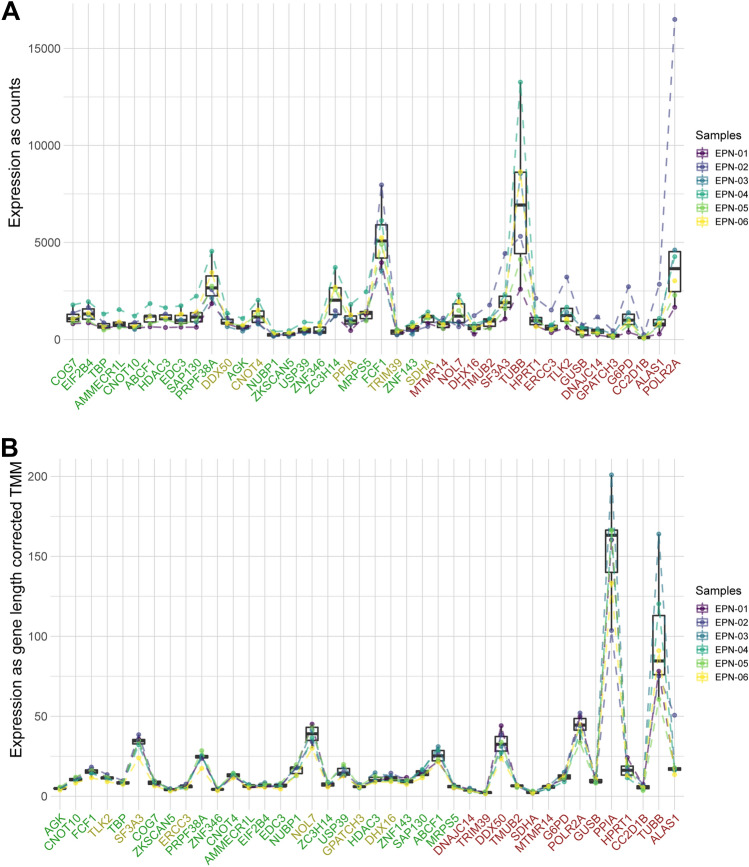
**A** Trend line of the forty housekeeping genes in NanoString samples. **B** Trend line of the forty housekeeping genes in RNA sequence samples. *Legend*: Green labels on the x-axis highlight the genes that were selected in both techniques, yellow is unique for the technique, and red means it was not selected for both techniques

The 40 assigned housekeeping genes in NanoString measurements were checked in the NanoString data. The twenty-four most stable housekeeping genes based on the expression ratio of the genes between all samples were selected by the geNorm algorithm [29] in the advanced analysis module of NanoString data (Fig. 1A). Applying the same algorithm to select the most stable housekeeping genes in the RNA sequence data resulted in a selection of 26 genes (Fig. 1B). Twenty housekeeping genes overlapped in the two selections.

### The detection level of the nCounter^®^ PanCancer Immune Profiling Panel

The 730 genes included in the nCounter^®^ PanCancer Immune Profiling Panel were examined in both datasets. From the 730 genes, 722 were identified in the RNA sequencing data. Interestingly, 7 of the 8 genes that were not identified in the RNA sequence data were above the detection limit in NanoString data. However, one gene (*KIR3DS1*) was not detected in NanoString nor identified in RNA sequencing (Table 3).

**Table 3:** NanoString gene expression values of the genes that were not identified with RNA sequencing

Gene symbol	Gene symbol NanoString	Alternative gene symbol	Average	Min	Max	Detected in # samples
*BAGE*	*BAGE*	*CT2.1, BAGE1*	52.7	Undetected	101.3	3
*HLA-DRB3*	*HLA-DRB3*	*HLA-DR3B*	11,030.93	1020.6	28,529.6	6
*HLA-DRB4*	*HLA-DRB4*	*DR4, DR-4, DRB4, HLA-DR4B*	779.0	Undetected	1399.1	3
*KIR3DS1*	*KIR_Activating_Subgroup_1*	*nkat10*	Undetected	Undetected	Undetected	0
*KIR2DS1*	*KIR_Activating_Subgroup_2*	*CD158H, EB6ActI, EB6ActII*	51.3	Undetected	51.3	1
*LTBR*	*LTBR*	*D12S370, TNFCR, TNFR-RP, TNFR2-RP, TNFR-III, TNFRSF3*	317.2	106.7	564.8	6
*MCAM*	*MCAM*	*MUC18, CD146, MelCAM, METCAM, HEMCAM*	431.1	103.2	941.8	6
*TARP*	*TARP*	*CD3G, TCRG, TCRGC1, TCRGC2*	110.7	47.1	301.3	5

### Comparability of NanoString and RNA sequence expression

**Fig. 2:**
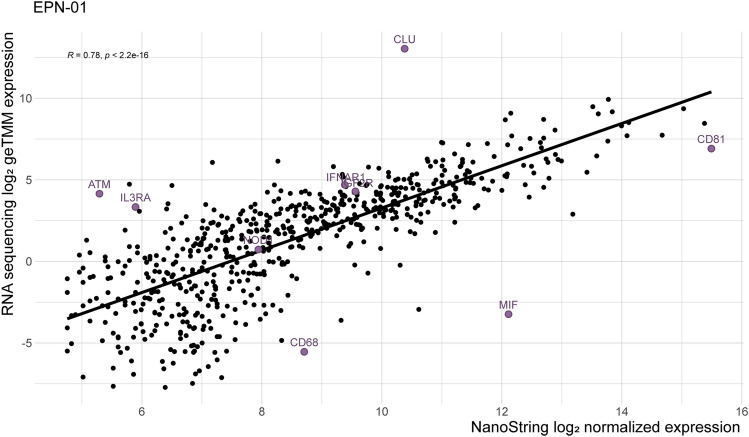
Pearson correlation of the genes that are above the detection limit and overlapping between both techniques in sample EPN-01. The three genes that are most divergent negatively (MIF, CD68, and CD81) and positively (CLU, ATM, and IL3RA) are highlighted next to the three genes that are the least divergent (IGF2R, NOD1, IFNAR1)

To determine the comparability between the two techniques, the Pearson correlation between genes that were above the detection limit was performed, and the correlation coefficient (*R*^2^) was calculated. EPN-01 is shown as an example in Fig. 2.

Interestingly, some genes like Clusterin (*CLU*), ATM Serine/Threonine Kinase (*ATM*), and Interleukin 3 Receptor Subunit Alpha (*IL3RA*) were detected at a higher level with the RNA sequence technique in all samples. On the other hand, Macrophage Migration Inhibitory Factor (MIF), Cluster of Differentiation 81 (*CD81*), Cluster of Differentiation 68 *(CD68),* and Cluster of Differentiation 81 (*CD81*) were detected at a higher level with the NanoString technique in all samples. The same results were confirmed by calculating the residuals, i.e., genes that fail to correlate, of the linear models per sample (Supplementary Fig. 2, Supplementary Table 1). The average Pearson correlation of the genes detected by NanoString and RNA sequencing was 0.82 (min. 0.78, max. 0.85), with an average *R*^2^ of 0.67 (Supplementary Figs. 3–7).

Dividing the 722 endogenous genes into bins based on their median expression from low to high showed an increased gene-specific inter-sample correlation for the highly expressed genes in both techniques. The average Spearman correlation was 0.3 across all 722 genes. In the NanoString data, 268 genes showed low expression (0–50 counts), 120 genes showed intermediate expression (50–200 counts) and 334 genes showed high expression (> 200). The Spearman correlation for the low expressed genes (average *R* = 0.13) is significantly lower compared to the intermediate (average *R* = 0.34, *p*-value < 0.0001) and high expressed genes (average *R* = 0.40, *p*-value < 0.0001). In the RNA sequence data, 261 genes showed low expression (0–1 count), 120 genes showed intermediate expression (1–3 counts) and 334 genes showed high expression (> 3 counts). The Spearman correlation for the low expressed genes (average *R* = 0.19, *p*-value < 0.0001) and intermediate expressed genes (average *R* = 0.27, *p*-value < 0.001) are significantly lower compared to the high expressed genes (average *R* = 0.39), Supplementary Fig. 8.

### The detection limit of nCounter in comparison to RNA sequencing

From the 722 identified genes (out of 730 genes) in both platforms, 464 genes were expressed above the detection limit and therefore measured by both methods in all samples. The gene *TLR9* was detected in four samples, but the expression levels were below the detection limit in the other two samples for both methods. The other 257 genes were in at least one sample detected by one method, but below the detection limit in the other of which four genes were only detected in all samples with RNA sequencing. There was not any gene only detected in all samples by NanoString (Supplementary Fig. 9).

### Comparison of the sensitivity of NanoString versus RNA sequencing

**Fig. 3:**
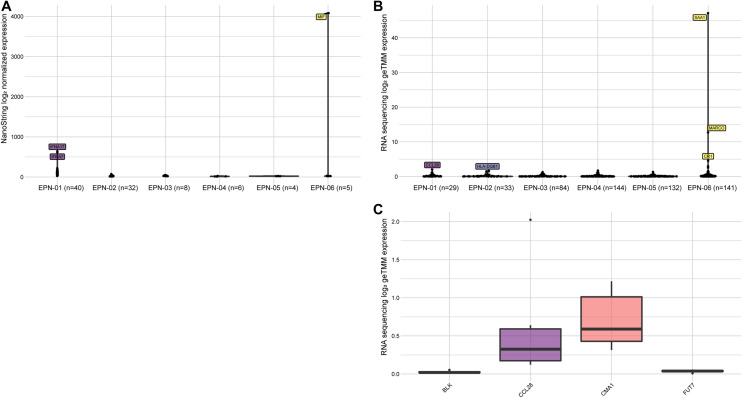
**A** NanoString gene counts of the genes per sample (*n* = the number of undetected genes) that were undetected by RNA sequencing. The highest expressed genes are highlighted. **B** RNA sequence counts of the genes per sample (*n* = the number of undetected genes) that were undetected by NanoString. The highest expressed genes are highlighted. **C** The RNA sequencing gene counts of the four genes were only detected by RNA sequencing in all samples (bottom)

To examine the sensitivity of both techniques, genes that were above the detection limit for only one technique were investigated. Sixty genes were detected by NanoString technology, but were under the detection limit by RNA sequencing in at least one sample. Remarkably, Macrophage Migration Inhibitory Factor (*MIF*) was detected with an expression value higher than 4000 counts in EPN-06 by the NanoString technique (Fig. 3A). On the contrary, 223 genes were only detected by RNA sequencing in at least one sample. The expression values from these genes vary from zero up to 28 (Fig. 3B). The expression of *BLK*, *CCL28*, *CMA1*, and *FUT7* was not detected in any sample with NanoString, but detected in all samples by RNA sequencing with expression values ranging from zero up to six (Fig. 3C).

### The identification of immune cells

**Fig. 4:**
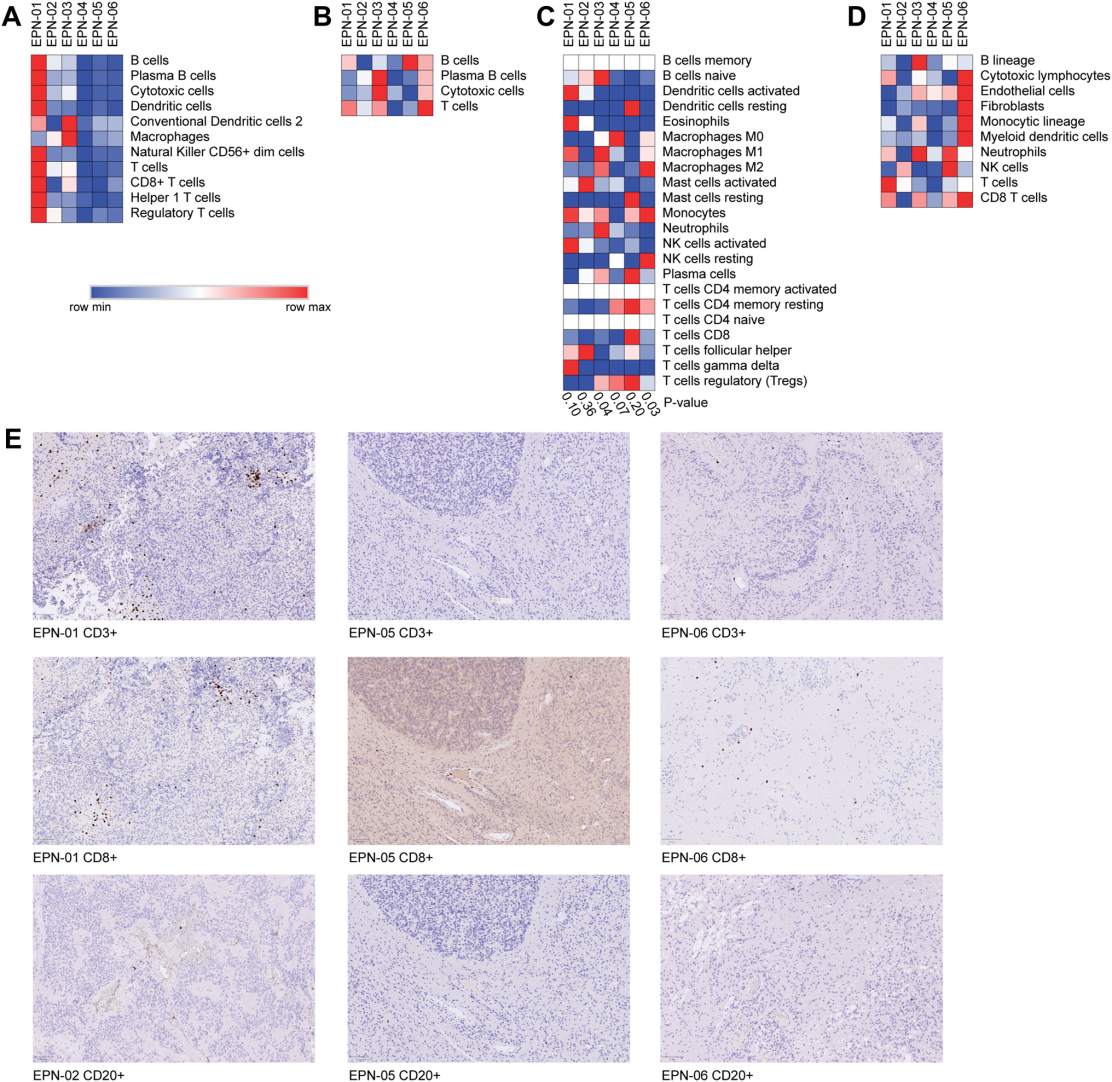
**A** Marker gene-based immune cell type abundances per sample with NanoString data. **B** Marker gene-based immune cell type abundances per sample with RNA sequence data. **C** CIBERSORT mixture-based immune cell abundances per sample in the RNA sequence data. **D** MCP-counter mixture-based immune cell abundances per sample in the RNA sequence data. **E** Immunohistochemistry staining of CD3 + (EPN-01, EPN-05, and EPN-06), CD8 + (EPN-01, EPN-05, and EPN-06), and CD20 + (EPN-02, EPN-05, and EPN-06) cells within the samples

Protein Tyrosine Phosphatase Receptor Type C (*PTPRC)* gene, also known as *CD45*, was above the detection limit in all samples by NanoString and RNA sequencing, which reflects leukocytes infiltrated into the ependymoma tumor (Supplementary Table 2). To define the type of immune cells that infiltrated the ependymoma samples measured with NanoString, the pairwise similarities for the 55 candidate marker genes were calculated. Genes that showed acceptable pairwise similarities (> 0.6) were selected as marker genes (Supplementary Document 1). In total, 25 genes were selected to identify 11 different immune cell types in ependymoma. These marker genes were then used to calculate the relative abundance per sample (Fig. 4A). The same method was used to identify immune cells in the RNA sequencing data. The marker gene-based method was investigated to identify immune cell types using RNA sequencing data. However, the pairwise similarities of most cells were below 0.6. (Supplementary Document 2). Therefore, the marker gene-based method was only applicable to a small set of immune cell types in the RNA sequencing data (Fig. 4B). The expression level of the selected marker genes was between 0 and 2.1 B cells, plasma B cells, cytotoxic cells, and T cells were identified using the two types of data. Nevertheless, both techniques show a different pattern of immune infiltration. The second method, CIBERSORT, was only applicable to RNA sequence data, as it expects an expression panel of 547 genes to be measured. This mixture-based method resulted in significant estimations for EPN-03 and EPN-06 (Fig. 4C). It showed infiltration of naïve b-cells, M1 macrophages, and neutrophils in EPN-03, whereas infiltration of M2 macrophages, monocytes, and resting NK cells was shown in EPN-06. A third method, MCP-counter, was used to determine the abundance of the immune cells based on the RNA sequence data. Similarly, MCP-counter expects a large set of 111 genes to be measured. Therefore, it was only applicable to RNA sequence data (Fig. 4D). MCP-counter showed the highest immune infiltration in EPN-06, but similarly to the gene maker method, a high infiltration of T cells was found in EPN-01. Immunohistochemistry showed infiltration of CD3 + cells in EPN-01, but CD3 + cells were not found in EPN-05 and EPN-06.

## Discussion

The characterization of the ependymoma’s TIME can be done with different techniques, each having its advantages and disadvantages. In that regard, the nCounter (PanCancer Immune Profiling Panel) of NanoString is compared to RNA sequencing to get a better understanding of these advantages and disadvantages. NanoString uses probes to measure up to 800 genes, whereas RNA sequencing is used to profile transcriptome-wide, including approximately 26,000 genes. RNA sequencing can be used to identify novel and rare transcripts like noncoding RNA and fusion RNA (Table 2). This makes RNA sequencing a suited tool for biomarker detection and measuring a broad scope of samples. The 40 housekeeping genes studied from both techniques showed a stable expression throughout all samples. The geNorm algorithm selected a similar number of housekeeping genes that were stable (Fig. 1). This shows that both methods are comparable regarding these housekeeping genes, which is to be expected. Eight out of the 730 endogenous genes of the PanCancer Immune Profiling Panel were not available in the RNA sequencing data (Table 3). This is most likely due to the complexity of the downstream analysis with RNA sequencing. The measurement of mRNA with RNA sequencing has FASTQ files as output, after which the downstream analysis is started including quality control, mapping, and normalization. Each of these steps can introduce errors and therefore experienced bioinformatics knowledge is needed. The complexity of the downstream analysis of RNA sequencing can cause important information to be lost. In particular, the quality control and mapping steps are tedious work. NanoString uses pre-defined panels with identifiers for each barcode detected; therefore, the processing of the measurements is less error-prone.

The significant Pearson correlations of the genes detected by both techniques (avg. Pearson correlation of 0.71) show, like the housekeeping genes, that comparable results can be found for most of the genes (Fig. 2). Nevertheless, there are discrepancies found between the two techniques. These discrepancies could be caused by the different types of samples used for each technique since FFPE samples were used for nCounter NanoString, whereas FF samples were used for RNA sequencing. Due to the frozen state of the FF samples, the RNA molecules are better preserved than in FFPE samples. The FF samples are recommended for RNA sequencing as high-quality RNA in a higher abundance is needed. On the other hand, PCR amplification can cause bias by amplifying specific genes. It is shown that the higher expressed genes have a more concordant expression between the two methods. The differences in the low-abundant genes can be due to the high sensitivity of the nCounter from NanoString by using barcodes of 100 bp, which makes it possible to measure low-abundant and lower-quality RNA more accurately even though FFPE samples were used [34]. In addition, RNA can only be measured with RNA sequencing after the RNA molecules undergo cDNA synthesis via reverse transcription of the mRNA and PCR amplification. In case the cDNA synthesis does not work properly the genes are less likely to be detected by RNA sequencing. The gene *ATM* (146,036 bases) was higher detected with RNA sequencing, whereas *CD68* (2621 bases) was higher detected with NanoString. This could be explained by the amplification bias in RNA sequencing. During PCR amplification, it is more likely to amplify a gene that has more reads present [35]. Nevertheless, this does not explain the different detection levels for *CLU* (17,784 bases) and *CD81* (21,221 bases). The 464 genes that were above the detection level in all samples confirm the comparability between the two techniques. The 257 genes that were detected by one method, but below the detection limit in the other in at least one sample, show the discrepancies that are caused by the technique or sample type. However, the RNA is expected to be more degraded in the FFPE samples sixty genes were detected by NanoString technology, but were under the detection limit by RNA sequencing in at least one sample with expression values higher than 4000 counts (Fig. 3A). That these genes are not detected with RNA sequencing could be due to the instability of the RNA molecule, as RNA sequencing needs stable reads over the whole exon region to detect the gene. The 223 genes that were only detected by RNA sequencing in at least one sample had an expression value ranging from zero up to 28 (Fig. 3B). *BLK*, *CCL28*, *CMA1*, and *FUT7* were not detected in any sample with NanoString, but detected in all samples by RNA sequencing with expression values ranging from zero up to six (Fig. 3C). These genes are most likely not detected by NanoString due to the degradation of the RNA molecule in FFPE.

The detection of *CD45* indicates that leukocytes infiltrated into the ependymoma tumor. Even though microglia are the most dominant immune cells in the brain (80%), other immune cell types have been identified in the brain including B cells, dendritic cells, macrophages, monocytes, myeloid cells, natural killer (NK) cells, and T cells [12, 36–38]. Nevertheless, the limited number of tumor-infiltrating immune cells compared to the high density of tumor cells can limit the sensitivity of bulk techniques [39]. The estimation of immune cell types in the samples by NanoString and RNA sequencing shows mostly incomparable results (Fig. 4). The marker gene method shows that the highest immune infiltration is in EPN-01 (Figure A), whereas the same method shows a higher immune infiltration in EPN-06 based on the RNA sequencing data. The low expression values in the RNA sequence data lead to low pairwise similarities and are therefore expected not to be accurate and are not suitable for most cell types using this method. The marker genes method cannot detect the absolute abundance of immune cells, but can only be used to describe the relative abundance between samples. The panel-based method CIBERSORT is only applicable to RNA sequencing results as it uses a large panel of genes that are not included in the standard panels of NanoString. CIBERSORT results in the absolute cell abundance which can be used to examine the relative abundance between samples. The RNA sequencing results include more cell types than estimated with the NanoString panel. Therefore, a fair comparison regarding the immune cell types cannot be made. MCP-counter is more comparable to the gene marker method used for NanoString. In the RNA sequencing data, both the marker gene method and MCP-counter describe the highest immune infiltration in EPN-06. On the other hand, based on the NanoString data analyzed with the marker gene method and the RNA sequencing data analyzed with the MCP-counter method show the highest infiltration of T cells in EPN-01. This has been confirmed with immunohistochemistry (Fig. 4E).

The number of samples is limited in this study. Nevertheless, the aim of this study was to highlight the (dis)concordance in the identification of the TIME in a cold tumor, like ependymomas, using gene expression data generated by the two most common techniques. Therefore, the variation in the included cohort of samples can be considered positive rather than negative because it covers a wider range of immune-cold tissue samples. The identification of immune cells is sample based. Therefore, the number of samples included to compare the two techniques has a limited effect on the presented results.

## Conclusion

In conclusion, both methods have their advantages and disadvantages. RNA sequencing is better suited for biomarker discovery, getting a broad overview of the samples regarding the TIME, detecting fusion genes, and mutation detection, although the low abundance genes might be missed out. The NanoString technique is easier to understand due to the simplified preprocessing steps both in the laboratory and dry lab. Furthermore, NanoString allows you to detect low-abundant genes in the panel of interest. The technique that was used to measure the samples had a considerable effect on the type of immune cells that were identified.

## Supplementary Information

Below is the link to the electronic supplementary material.

Supplementary file1 (DOCX 2521 KB)

Supplementary file2 (XLSX 76 KB)

Supplementary file3 (CSV 0 KB)

Supplementary file4 (DOCX 69 KB)

Supplementary file5 (DOCX 70 KB)

## Data Availability

The NanoString data presented in the study are deposited in the Gene Expression Omnibus (GEO) repository, accession number GSE216478. The RNA sequencing data presented in the study are deposited in the European Genome-phenome Archive (EGA) repository, accession number EGAS00001006535.
