# Supplementary material for: Characterizing the tumor immune microenvironment of ependymomas using targeted gene expression profiles and RNA sequencing
Source: Cancer Immunol Immunother. 2023 Apr 19;72(8):2659–70. doi: 10.1007/s00262-023-03450-2 (PMC10361846; doi:10.1007/s00262-023-03450-2)
Supplement: Supplementary file 1 — Supplementary file1 (DOCX 2521 KB) [file 262_2023_3450_MOESM1_ESM.docx]

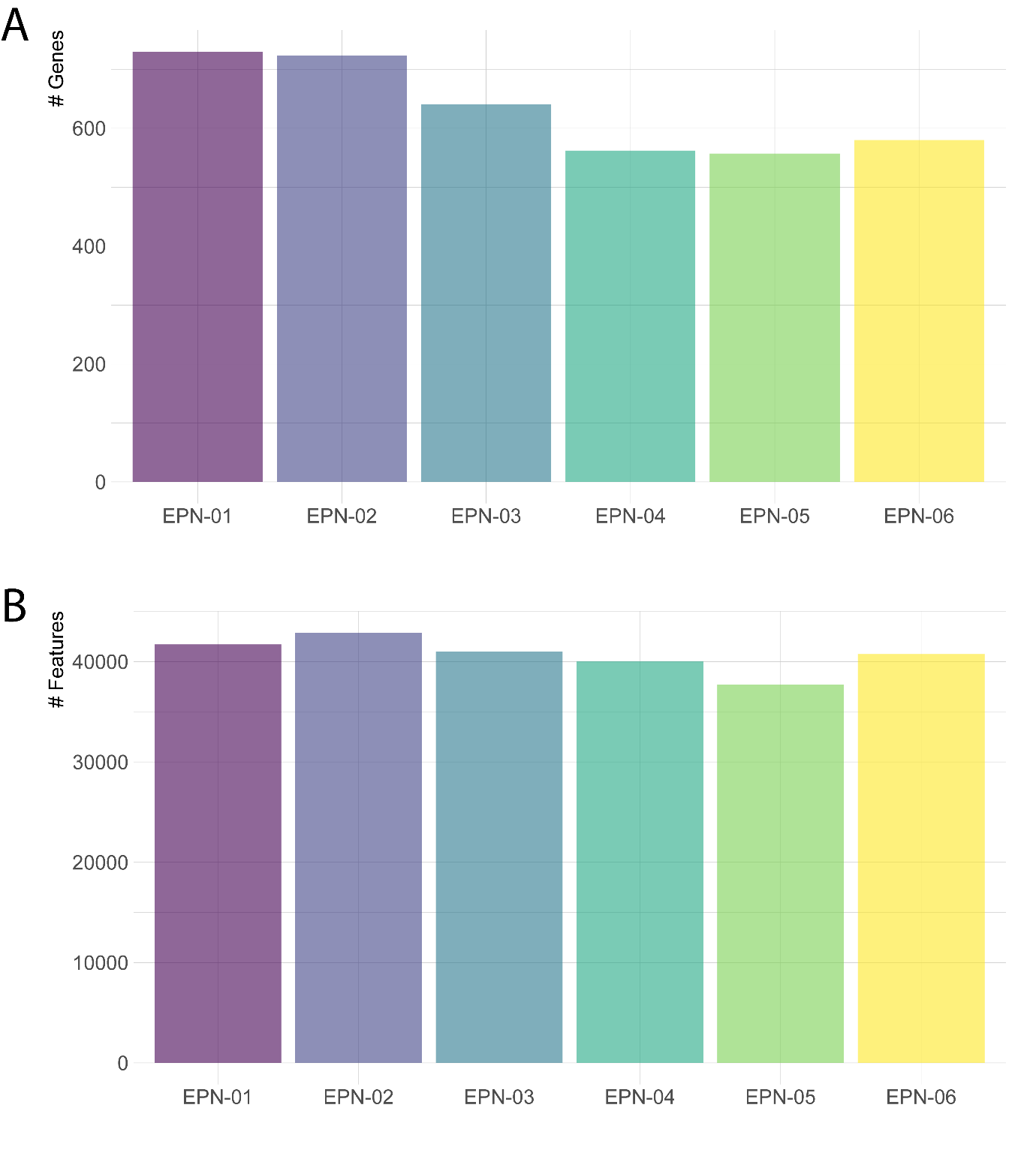


Supplementary Figure 1 **(A)** The total number of genes detected by the nCounter NanoString Immune profiling panel per sample. **(B)** The total number of features detected by RNA-sequencing per sample.


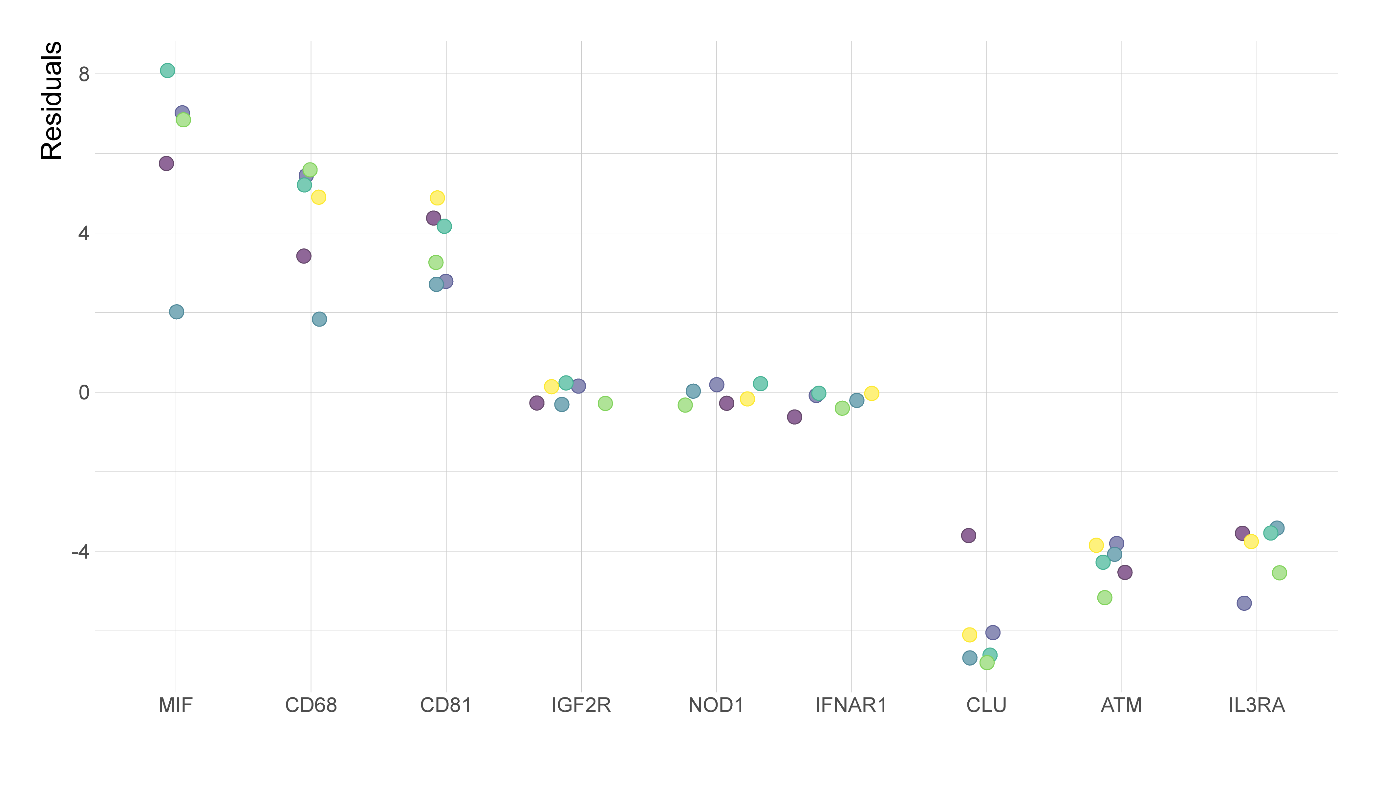


Supplementary Figure 2 The residuals, i.e., the vertical distance between the gene expression and the regression line of the three genes that are most divergent negatively (MIF, CD68 and CD81) and positively (CLU, ATM and IL3RA) are shown next to the three genes that are the least divergent (IGF2R, NOD1, IFNAR1).


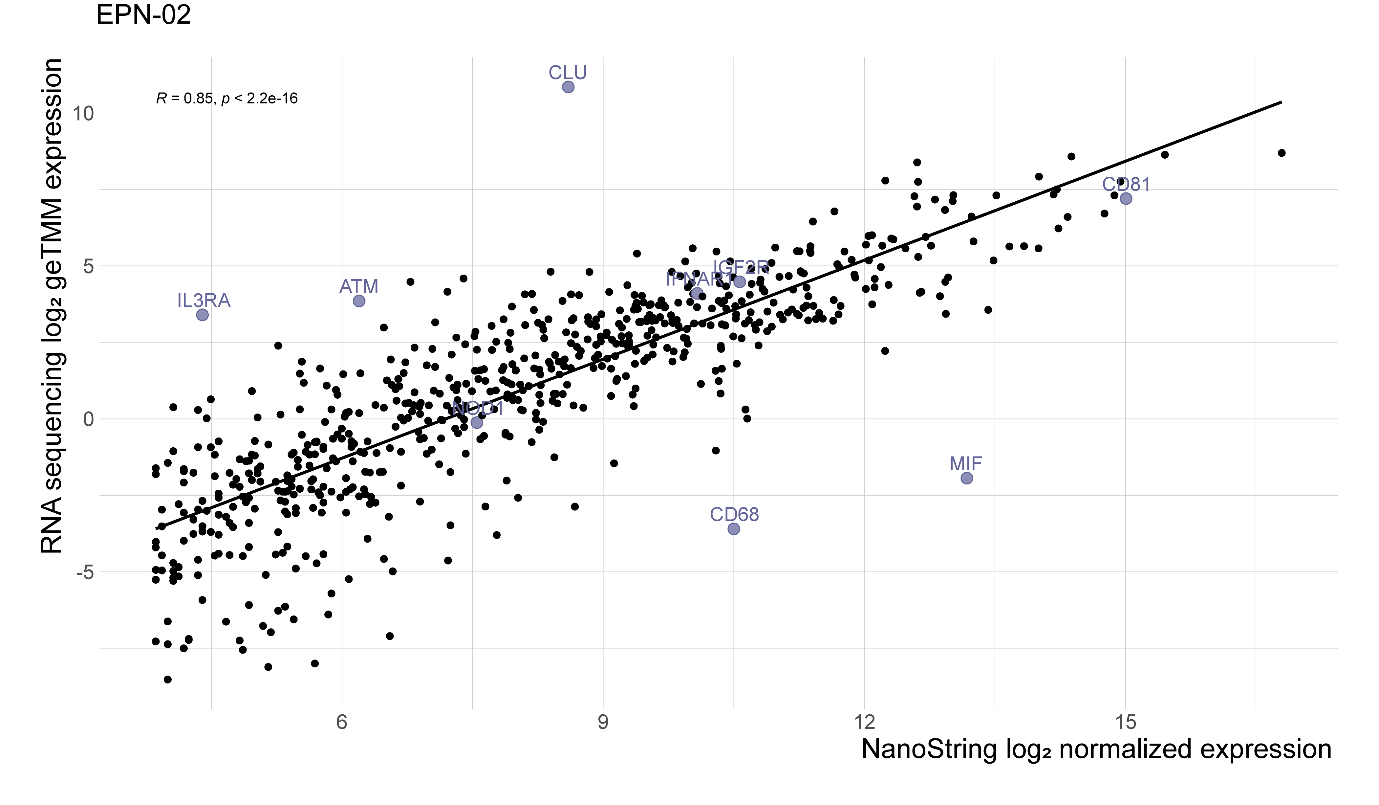


Supplementary Figure 3 Pearson correlation of the genes that are above the detection limit and overlapping between both techniques in sample EPN-02. The three genes that are most divergent negatively (MIF, CD68 and CD81) and positively (CLU, ATM and IL3RA) are highlighted next to the three genes that are the least divergent (IGF2R, NOD1, IFNAR1).


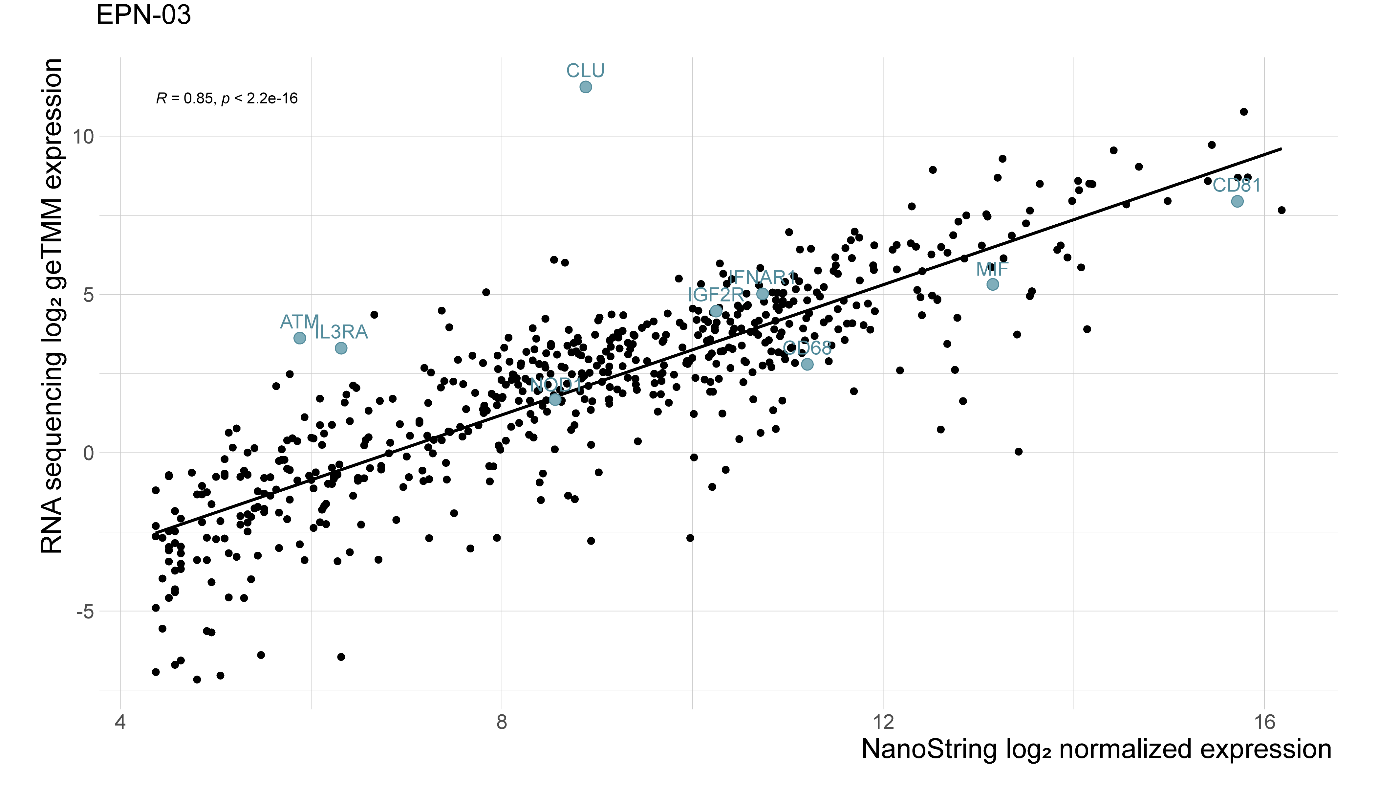


Supplementary Figure 4 Pearson correlation of the genes that are above the detection limit and overlapping between both techniques in sample EPN-03. The three genes that are most divergent negatively (MIF, CD68 and CD81) and positively (CLU, ATM and IL3RA) are highlighted next to the three genes that are the least divergent (IGF2R, NOD1, IFNAR1).


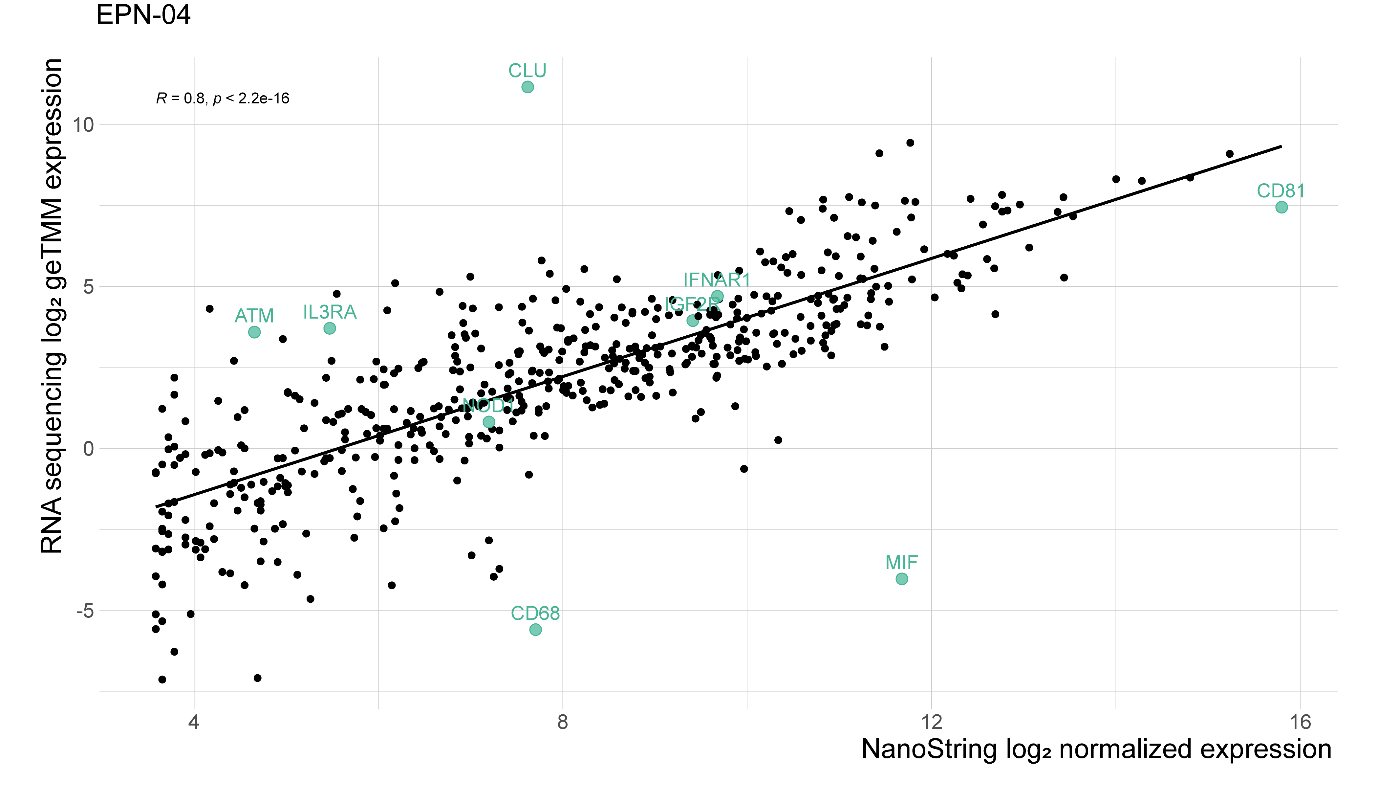


Supplementary Figure 5 Pearson correlation of the genes that are above the detection limit and overlapping between both techniques in sample EPN-04. The three genes that are most divergent negatively (MIF, CD68 and CD81) and positively (CLU, ATM and IL3RA) are highlighted next to the three genes that are the least divergent (IGF2R, NOD1, IFNAR1).


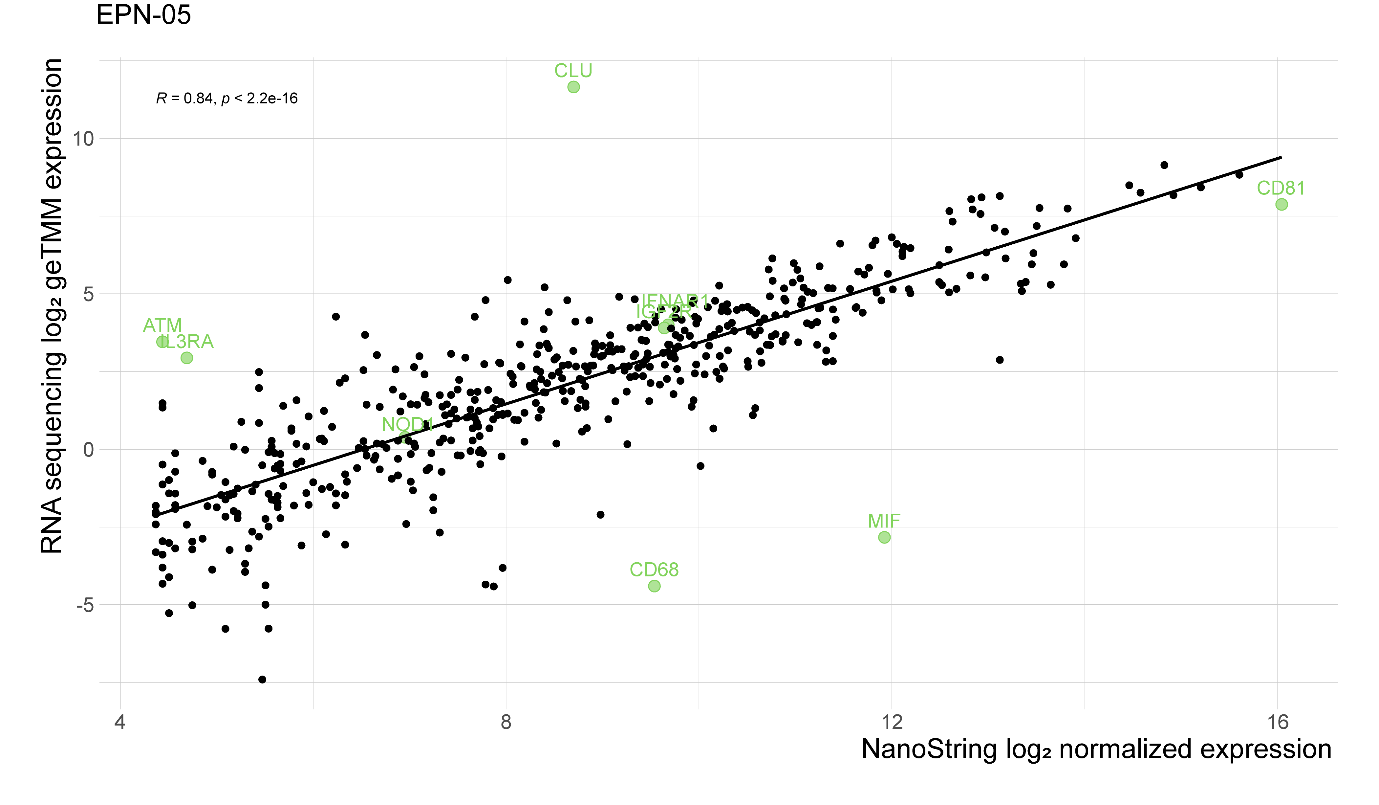


Supplementary Figure 6 Pearson correlation of the genes that are above the detection limit and overlapping between both techniques in sample EPN-05. The three genes that are most divergent negatively (MIF, CD68 and CD81) and positively (CLU, ATM and IL3RA) are highlighted next to the three genes that are the least divergent (IGF2R, NOD1, IFNAR1).


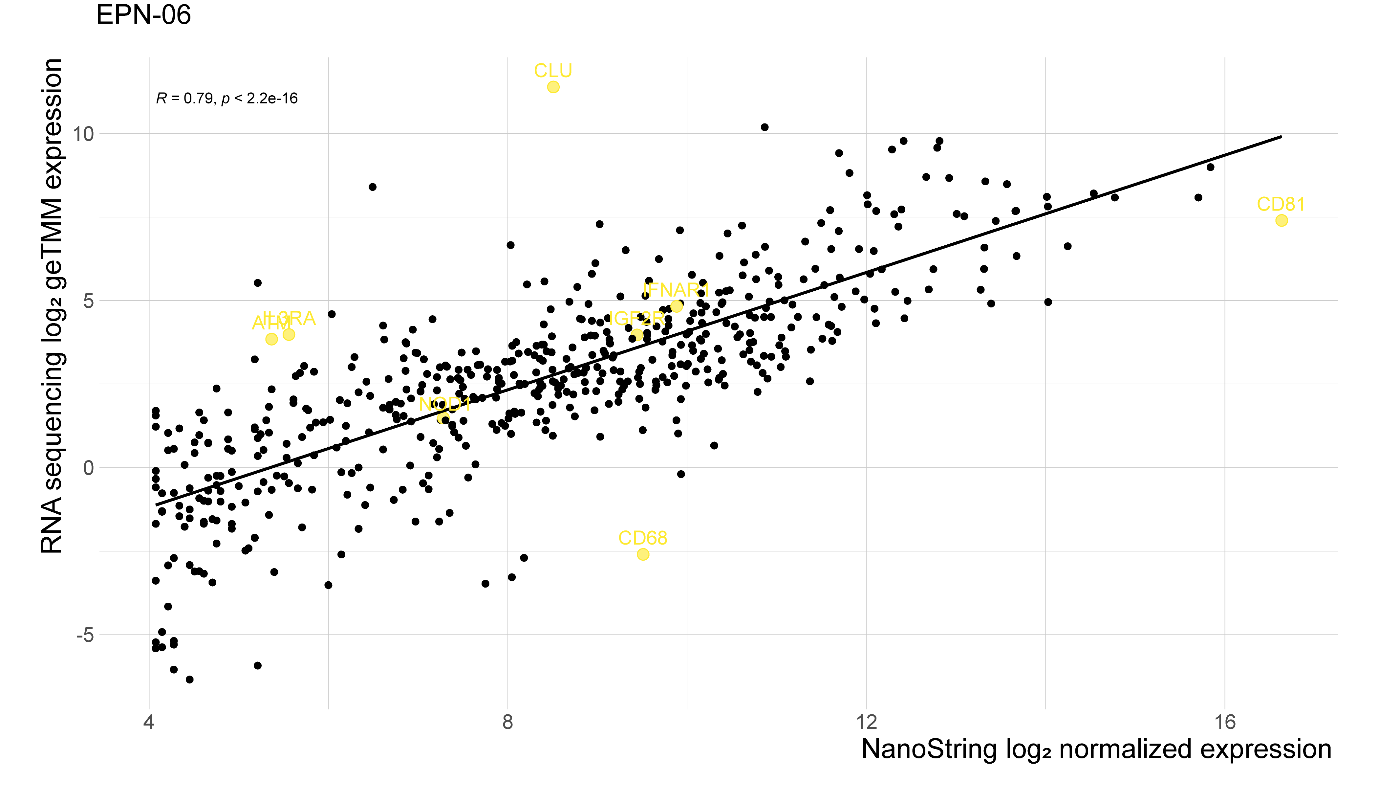


Supplementary Figure 7 Pearson correlation of the genes that are above the detection limit and overlapping between both techniques in sample EPN-06. The three genes that are most divergent negatively (MIF, CD68 and CD81) and positively (CLU, ATM and IL3RA) are highlighted next to the three genes that are the least divergent (IGF2R, NOD1, IFNAR1).


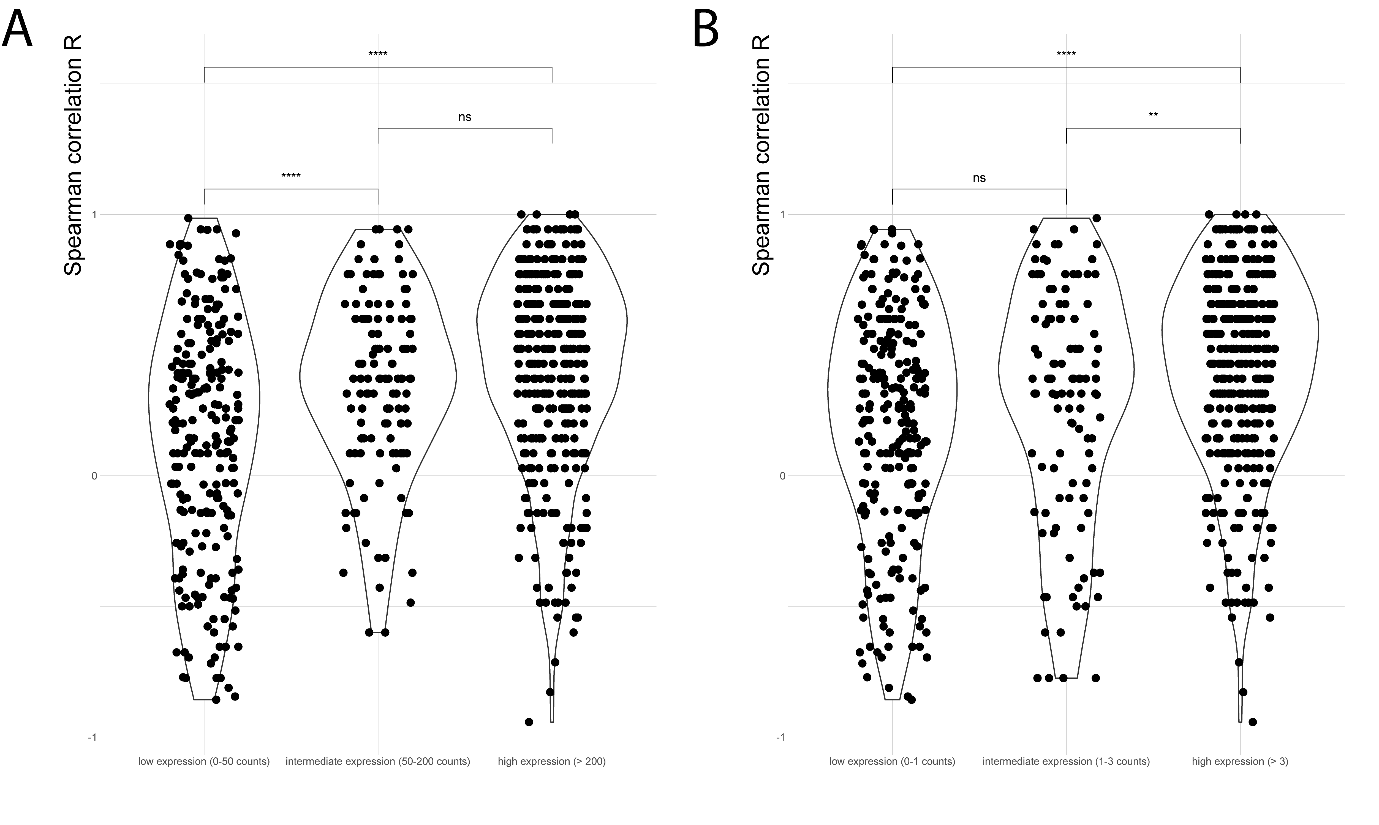


Supplementary Figure 8 **(A)** The gene-specific inter-sample Spearman correlation for the 268 genes that showed low expression (0-50 counts), 120 genes that showed intermediate expression (50-200 counts), and 334 genes that showed high expression (> 200) in the NanoString data. **(B)** The gene-specific inter-sample Spearman correlation for the 261 genes that showed low expression (0-1 counts), 120 genes that showed intermediate expression (1-3 counts), and 334 genes that showed high expression (> 3) in the RNA-sequence data.


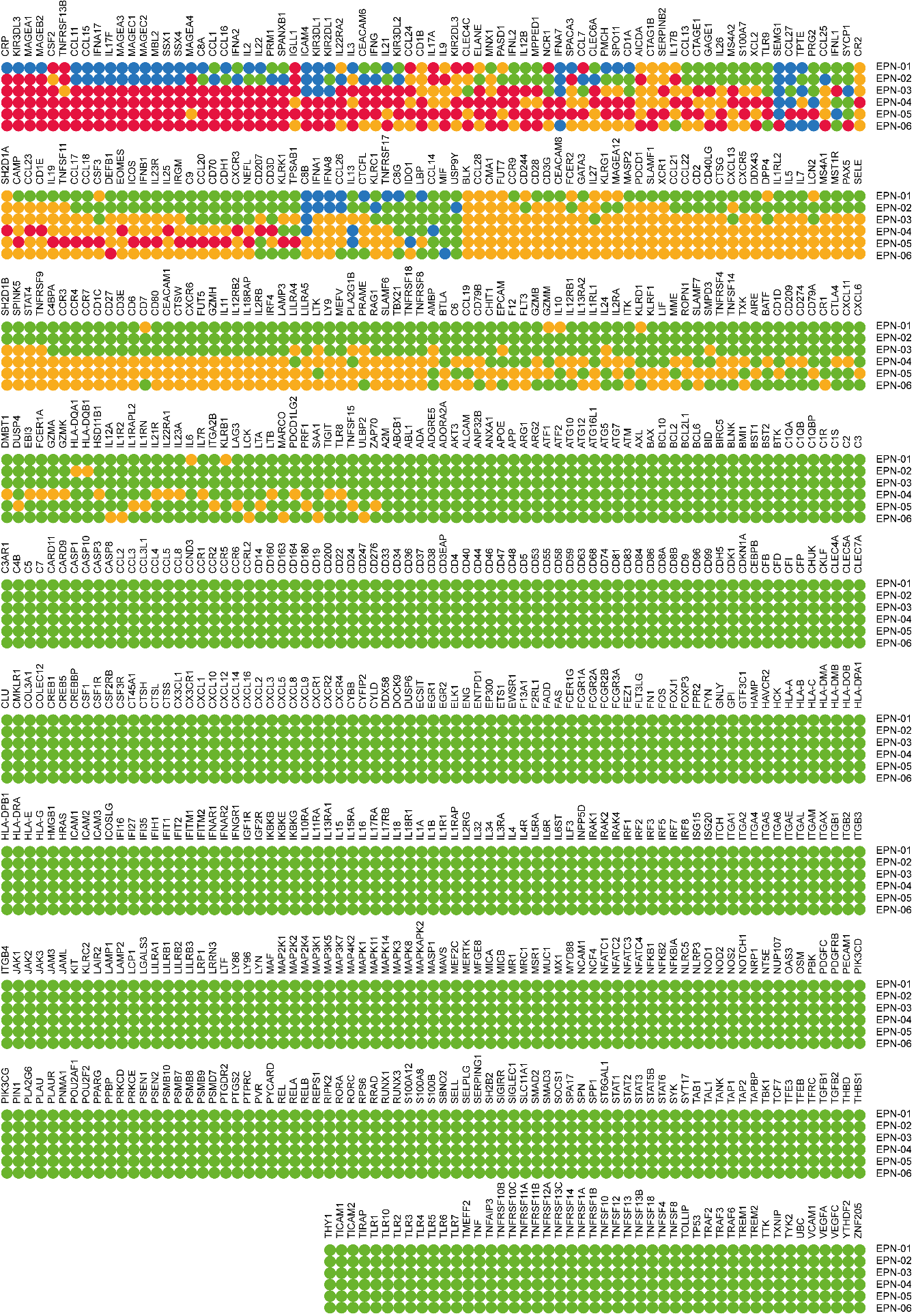


Supplementary Figure 9 The 722 identified genes. 464 genes were expressed above detection limit and therefore measured by both methods (green) in all samples (6x green). The gene TLR9 was detected in four samples by both methods, but the expression levels were below detection limit by both methods (red) in the other two samples (4x green, 2x red). The other 257 genes were in at least one sample detected by one method (only NanoString is blue, only RNA sequencing is yellow), but below detection limit in the other of which four genes were only detected in all samples with RNA sequencing (6x yellow).
