# Supplementary material for: Characterizing the tumor immune microenvironment of ependymomas using targeted gene expression profiles and RNA sequencing
Source: Cancer Immunol Immunother. 2023 Apr 19;72(8):2659–70. doi: 10.1007/s00262-023-03450-2 (PMC10361846; doi:10.1007/s00262-023-03450-2)
Supplement: Supplementary file 4 — Supplementary file4 (DOCX 69 KB) [file 262_2023_3450_MOESM4_ESM.docx]

# Supplementary Document 1: NanoString cell definitions

To characterize the immune cell infiltration with the NanoString data, gene markers were identified. The gene markers were selected by calculating the pairwise similarity between all pairs of candidate marker genes that were **above the detection limit in at least 50% of the samples**. The gene pairs with a **pairwise similarity above 0.6** were selected to describe the immune cells. Each immune cell type needed at least two unique genes. This method is further described in de Koning et al., 2021.

de Koning, W., Latifi, D., Li, Y., van Eijck, C. H. J., Stubbs, A. P., & Mustafa, D. A. M. (2021). Identification, Validation, and Utilization of Immune Cells in Pancreatic Ductal Adenocarcinoma Based on Marker Genes. *Frontiers in Immunology*, *12*. https://doi.org/10.3389/fimmu.2021.649061

Table of Contents

[Supplementary Document 1: NanoString cell definitions 1](#_Toc117092707)

[Result table 3](#_Toc117092708)

[B cells 4](#_Toc117092709)

[Plasma B cells 4](#_Toc117092710)

[Regulatory B cells 5](#_Toc117092711)

[Cytotoxic cells 5](#_Toc117092712)

[Dendritic cells 6](#_Toc117092713)

[Conventional Dendritic cells 1 6](#_Toc117092714)

[Conventional Dendritic cells 2 6](#_Toc117092715)

[Macrophages 7](#_Toc117092716)

[Antigen presenting cells 7](#_Toc117092717)

[M2 Macrophages 7](#_Toc117092718)

[Mast cells 8](#_Toc117092719)

[Monocytes 8](#_Toc117092720)

[Natural Killer cells 8](#_Toc117092721)

[Natural Killer CD56+ dim cells 8](#_Toc117092722)

[Neutrophils 9](#_Toc117092723)

[T cells 9](#_Toc117092724)

[CD4+ T cells 9](#_Toc117092725)

[CD8+ T cells 10](#_Toc117092726)

[Exhausted CD8+ T cells 10](#_Toc117092727)

[Helper 1 T cells 10](#_Toc117092728)

[Regulatory T cells 11](#_Toc117092729)

[CD45+ 11](#_Toc117092730)

# Result table

| **Cell type** | **Candidate marker genes** | **Selected markers** |
| --- | --- | --- |
| B cells | *BLK, CD19, CD22, CR2, MS4A1* | *CD19, CD22, MS4A1* |
| Plasma B cells | *CD27, CD38, SLAMF7, TNFRSF17* | *CD27, SLAMF7, TNFRSF17* |
| Regulatory B cells | *CD1D, CD5* | *-* |
| Cytotoxic cells | *GZMA, GZMB, GZMH, KLRB1, KLRD1, KLRK1, PRF1* | *GZMA, GZMB, GZMH, KLRK1* |
| Dendritic cells | *CD1A, CD1C* | *CD1A, CD1C* |
| Conventional Dendritic cells 1 | *BTLA, XCR1* | *-* |
| Conventional Dendritic cells 2 | *ITGAM, ITGAX* | *ITGAM, ITGAX* |
| Macrophages | *CD68, FCGR2A* | *CD68, FCGR2A* |
| Antigen presenting cells | *CD80, CD86* | *-* |
| M2 Macrophages | *CD163, MRC1* | *-* |
| Mast cells | *MS4A2, TPSAB1* | *-* |
| Monocytes | *CD14, CD33, TLR2* | *-* |
| Natural Killer cells | *NCR1* | *-* |
| Natural Killer CD56+ dim cells | *KIR3DL1* | *KIR3DL1* |
| Neutrophils | *CSF3R, FCGR3A* | *-* |
| T cells | *CD3D, CD3E, CD3G, CD6, SH2D1A* | *CD3D, CD3E, CD6* |
| CD4+ T cells | *CD4, SELL* | *-* |
| CD8+ T cells | *CD8A, CD8B* | *CD8A, CD8B* |
| Exhausted CD8+ T cells | *LAG3, PDCD1, TIGIT* | *-* |
| Helper 1 T cells | *TBX21* | *TBX21* |
| Regulatory T cells | *FOXP3, IL2RA* | *FOXP3, IL2RA* |
| CD45+ | *PTPRC* | *PTPRC* |

## B cells

Candidates: *BLK, CD19, CD22, CR2, MS4A1*

Selected: *CD19, CD22, MS4A1*

Detection limit (DL):

| **Gene** | **Below DL** | **Above DL** |
| --- | --- | --- |
| *BLK* | 6 | 0 |
| *CD19* | 0 | 6 |
| *CD22* | 0 | 6 |
| *CR2* | 6 | 0 |
| *MS4A1* | 4 | 2 |

Pairwise similarity matrix ALL samples:

|  | *BLK* | *CD19* | *CD22* | *CR2* | *MS4A1* |
| --- | --- | --- | --- | --- | --- |
| *BLK* | 1.0 | 0.2 | 0.1 | 0.5 | 0.3 |
| *CD19* | 0.2 | 1.0 | 0.9 | 0.2 | 0.7 |
| *CD22* | 0.1 | 0.9 | 1.0 | 0.1 | 0.5 |
| *CR2* | 0.5 | 0.2 | 0.1 | 1.0 | 0.5 |
| *MS4A1* | 0.3 | 0.7 | 0.5 | 0.5 | 1.0 |

## Plasma B cells

Candidates: *CD27, CD38, SLAMF7, TNFRSF17*

Selected: *CD27, SLAMF7, TNFRSF17*

Detection limit:

| **Gene** | **Below DL** | **Above DL** |
| --- | --- | --- |
| *CD27* | 3 | 3 |
| *CD38* | 0 | 6 |
| *SLAMF7* | 2 | 4 |
| *TNFRSF17* | 3 | 3 |

Pairwise similarity matrix ALL samples:

|  | *CD27* | *CD38* | *SLAMF7* | *TNFRSF17* |
| --- | --- | --- | --- | --- |
| *CD27* | 1 | 0.3 | 0.9 | 0.8 |
| *CD38* | 0.3 | 1 | 0.5 | 0.5 |
| *SLAMF7* | 0.9 | 0.5 | 1 | 0.9 |
| *TNFRSF17* | 0.8 | 0.5 | 0.9 | 1 |

## Regulatory B cells

Candidates: *CD1D, CD5*

Selected:

Detection limit:

| **Gene** | **Below DL** | **Above DL** |
| --- | --- | --- |
| *CD1D* | 1 | 5 |
| *CD5* | 0 | 6 |

Pairwise similarity matrix ALL samples:

|  | *CD1D* | *CD5* |
| --- | --- | --- |
| *CD1D* | 1 | 0.2 |
| *CD5* | 0.2 | 1 |

## Cytotoxic cells

Candidates: *GZMA, GZMB, GZMH, KLRB1, KLRD1, KLRK1, PRF1*

Selected: *GZMA, GZMB, GZMH, KLRK1*

Detection limit:

| **Gene** | **Below DL** | **Above DL** |
| --- | --- | --- |
| *GZMA* | 1 | 5 |
| *GZMB* | 2 | 4 |
| *GZMH* | 3 | 3 |
| *KLRB1* | 1 | 5 |
| *KLRD1* | 2 | 4 |
| *KLRK1* | 2 | 4 |
| *PRF1* | 1 | 5 |

Pairwise similarity matrix ALL samples:

|  | *GZMA* | *GZMB* | *GZMH* | *KLRB1* | *KLRD1* | *KLRK1* | *PRF1* |
| --- | --- | --- | --- | --- | --- | --- | --- |
| *GZMA* | 1.0 | 0.7 | 0.8 | 0.3 | 0.0 | 0.7 | 0.7 |
| *GZMB* | 0.7 | 1.0 | 0.8 | -0.1 | 0.1 | 0.6 | 0.5 |
| *GZMH* | 0.8 | 0.8 | 1.0 | -0.1 | 0.0 | 0.9 | 0.3 |
| *KLRB1* | 0.3 | -0.1 | -0.1 | 1.0 | -0.1 | 0.0 | 0.1 |
| *KLRD1* | 0.0 | 0.1 | 0.0 | -0.1 | 1.0 | 0.0 | 0.0 |
| *KLRK1* | 0.7 | 0.6 | 0.9 | 0.0 | 0.0 | 1.0 | 0.1 |
| *PRF1* | 0.7 | 0.5 | 0.3 | 0.1 | 0.0 | 0.1 | 1.0 |

## Dendritic cells

Candidates: *CD1A, CD1C*

Selected: *CD1A, CD1C*

Detection limit:

| **Gene** | **Below DL** | **Above DL** |
| --- | --- | --- |
| *CD1A* | 3 | 3 |
| *CD1C* | 3 | 3 |

Pairwise similarity matrix ALL samples:

|  | *CD1A* | *CD1C* |
| --- | --- | --- |
| *CD1A* | 1 | 1 |
| *CD1C* | 1 | 1 |

## Conventional Dendritic cells 1

Candidates: *BTLA, XCR1*

Selected:

Detection limit:

| **Gene** | **Below DL** | **Above DL** |
| --- | --- | --- |
| *BTLA* | 2 | 4 |
| *XCR1* | 5 | 1 |

Pairwise similarity matrix ALL samples:

|  | *BTLA* | *XCR1* |
| --- | --- | --- |
| *BTLA* | 1 | 0.3 |
| *XCR1* | 0.3 | 1 |

## Conventional Dendritic cells 2

Candidates: *ITGAM, ITGAX*

Selected: *ITGAM, ITGAX*

Detection limit:

| **Gene** | **Below DL** | **Above DL** |
| --- | --- | --- |
| *ITGAM* | 0 | 6 |
| *ITGAX* | 0 | 6 |

Pairwise similarity matrix ALL samples:

|  | *ITGAM* | *ITGAX* |
| --- | --- | --- |
| *ITGAM* | 1 | 0.8 |
| *ITGAX* | 0.8 | 1 |

## Macrophages

Candidates: *CD68, FCGR2A*

Selected: *CD68, FCGR2A*

Detection limit:

| **Gene** | **Below DL** | **Above DL** |
| --- | --- | --- |
| *CD68* | 0 | 6 |
| *FCGR2A* | 0 | 6 |

Pairwise similarity matrix ALL samples:

|  | *CD68* | *FCGR2A* |
| --- | --- | --- |
| *CD68* | 1 | 0.9 |
| *FCGR2A* | 0.9 | 1 |

## Antigen presenting cells

Candidates: *CD80, CD86*

Selected:

Detection limit:

| **Gene** | **Below DL** | **Above DL** |
| --- | --- | --- |
| *CD80* | 3 | 3 |
| *CD86* | 0 | 6 |

Pairwise similarity matrix ALL samples:

|  | *CD80* | *CD86* |
| --- | --- | --- |
| *CD80* | 1 | 0.5 |
| *CD86* | 0.5 | 1 |

## M2 Macrophages

Candidates: *CD163, MRC1*

Selected:

Detection limit:

| **Gene** | **Below DL** | **Above DL** |
| --- | --- | --- |
| *CD163* | 0 | 6 |
| *MRC1* | 0 | 6 |

Pairwise similarity matrix ALL samples:

|  | *CD163* | *MRC1* |
| --- | --- | --- |
| *CD163* | 1 | 0.1 |
| *MRC1* | 0.1 | 1 |

## Mast cells

Candidates: *MS4A2, TPSAB1*

Selected:

Detection limit:

| **Gene** | **Below DL** | **Above DL** |
| --- | --- | --- |
| *MS4A2* | 4 | 2 |
| *TPSAB1* | 2 | 4 |

Pairwise similarity matrix ALL samples:

|  | *MS4A2* | *TPSAB1* |
| --- | --- | --- |
| *MS4A2* | 1.0 | 0.0 |
| *TPSAB1* | 0.0 | 1.0 |

## Monocytes

Candidates: *CD14, CD33, TLR2*

Selected:

Detection limit:

| **Gene** | **Below DL** | **Above DL** |
| --- | --- | --- |
| *CD14* | 0 | 6 |
| *CD33* | 0 | 6 |
| *TLR2* | 0 | 6 |

Pairwise similarity matrix ALL samples:

|  | *CD14* | *CD33* | *TLR2* |
| --- | --- | --- | --- |
| *CD14* | 1.0 | 0.0 | 0.2 |
| *CD33* | 0.0 | 1.0 | 0.1 |
| *TLR2* | 0.2 | 0.1 | 1.0 |

## Natural Killer cells

Candidates: *NCR1*

Selected:

Detection limit:

| **Gene** | **Below DL** | **Above DL** |
| --- | --- | --- |
| *NCR1* | 4 | 2 |

## Natural Killer CD56+ dim cells

Candidates: *KIR3DL1*

Selected: *KIR3DL1*

Detection limit:

| **Gene** | **Below DL** | **Above DL** |
| --- | --- | --- |
| *KIR3DL1* | 3 | 3 |

## Neutrophils

Candidates: *CSF3R, FCGR3A*

Selected:

Detection limit:

| **Gene** | **Below DL** | **Above DL** |
| --- | --- | --- |
| *CSF3R* | 0 | 6 |
| *FCGR3A* | 0 | 6 |

Pairwise similarity matrix ALL samples:

|  | *CSF3R* | *FCGR3A* |
| --- | --- | --- |
| *CSF3R* | 1 | 0.2 |
| *FCGR3A* | 0.2 | 1 |

## T cells

Candidates: *CD3D, CD3E, CD3G, CD6, SH2D1A*

Selected: *CD3D, CD3E, CD6*

Detection limit:

| **Gene** | **Below DL** | **Above DL** |
| --- | --- | --- |
| *CD3D* | 2 | 4 |
| *CD3E* | 3 | 3 |
| *CD3G* | 5 | 1 |
| *CD6* | 3 | 3 |
| *SH2D1A* | 6 | 0 |

Pairwise similarity matrix ALL samples:

|  | *CD3D* | *CD3E* | *CD3G* | *CD6* | *SH2D1A* |
| --- | --- | --- | --- | --- | --- |
| *CD3D* | 1 | 0.8 | 0.8 | 0.8 | 0.2 |
| *CD3E* | 0.8 | 1 | 0.7 | 0.8 | 0.4 |
| *CD3G* | 0.8 | 0.7 | 1 | 1 | 0.3 |
| *CD6* | 0.8 | 0.8 | 1 | 1 | 0.3 |
| *SH2D1A* | 0.2 | 0.4 | 0.3 | 0.3 | 1 |

## CD4+ T cells

Candidates: *CD4, SELL*

Selected:

Detection limit:

| **Gene** | **Below DL** | **Above DL** |
| --- | --- | --- |
| *CD4* | 0 | 6 |
| *SELL* | 0 | 6 |

Pairwise similarity matrix ALL samples:

|  | *CD4* | *SELL* |
| --- | --- | --- |
| *CD4* | 1 | 0.5 |
| *SELL* | 0.5 | 1 |

## CD8+ T cells

Candidates: *CD8A, CD8B*

Selected: *CD8A, CD8B*

Detection limit:

| **Gene** | **Below DL** | **Above DL** |
| --- | --- | --- |
| *CD8A* | 0 | 6 |
| *CD8B* | 0 | 6 |

Pairwise similarity matrix ALL samples:

|  | *CD8A* | *CD8B* |
| --- | --- | --- |
| *CD8A* | 1 | 0.8 |
| *CD8B* | 0.8 | 1 |

## Exhausted CD8+ T cells

Candidates: *LAG3, PDCD1, TIGIT*

Selected:

Detection limit:

| **Gene** | **Below DL** | **Above DL** |
| --- | --- | --- |
| *LAG3* | 1 | 5 |
| *PDCD1* | 5 | 1 |
| *TIGIT* | 1 | 5 |

Pairwise similarity matrix ALL samples:

|  | *LAG3* | *PDCD1* | *TIGIT* |
| --- | --- | --- | --- |
| *LAG3* | 1 | 0.4 | 0.4 |
| *PDCD1* | 0.4 | 1 | 0.3 |
| *TIGIT* | 0.4 | 0.3 | 1 |

## Helper 1 T cells

Candidates: *TBX21*

Selected: *TBX21*

Detection limit:

| **Gene** | **Below DL** | **Above DL** |
| --- | --- | --- |
| *TBX21* | 3 | 3 |

## Regulatory T cells

Candidates: *FOXP3, IL2RA*

Selected: *FOXP3, IL2RA*

Detection limit:

| **Gene** | **Below DL** | **Above DL** |
| --- | --- | --- |
| *FOXP3* | 0 | 6 |
| *IL2RA* | 2 | 4 |

Pairwise similarity matrix ALL samples:

|  | *FOXP3* | *IL2RA* |
| --- | --- | --- |
| *FOXP3* | 1 | 0.7 |
| *IL2RA* | 0.7 | 1 |

## CD45+

Candidates: *PTPRC*

Selected:

Detection limit:

| **Gene** | **Below DL** | **Above DL** |
| --- | --- | --- |
| *PTPRC* | 0 | 6 |
